# Supplementary material for: Continuous, Low Latency Estimation of the Size and Shape of Single Proteins from Real-Time Nanopore Data
Source: Anal Chem. 2025 Dec 31;98(1):224–34. doi: 10.1021/acs.analchem.5c04044 (PMC12809640; doi:10.1021/acs.analchem.5c04044)
Supplement: Supplementary file 1 [file ac5c04044_si_001.pdf]

Supporting Information for

Continuous, Low Latency Estimation of the Size and Shape of Single Proteins from Real-Time Nanopore Data

Yuanjie Li[1], Cuifeng Ying[2], Michael Mayer[1*]

[1] Adolphe Merkle Institute, University of Fribourg, Chemin des Verdiers 4, CH-1700 Fribourg, Switzerland

[2] Advanced Optics and Photonics Laboratory, Department of Engineering, School of Science and Technology, Nottingham Trent University, Nottingham NG118 NS, United Kingdom

*Corresponding Author Email: michael.mayer@unifr.ch

Table of Contents

Supplementary Note 1. Algorithms for calculating mean, median, standard deviation, minimum, and maximum in the sliding windows S3

Supplementary Note 2. Simulation of protein translocation through nanopores S4

Supplementary Note 3. Naïve Bayes classifier for classification of protein mixtures S8

Supplementary Note 4. Effects of baseline modulation on the determination of protein shape and volume S9

Figure S1. Screenshot of the data acquisition and real-time estimation of protein using PyDAQ S10

Figure S2. Example current trace demonstrating the changing of internal variables calculated by the TSW algorithm during the detection of a resistive pulse S11

Figure S3. Effects of *n-sigma* parameter and signal-to-noise ratio of resistive pulses on the performance of the TSW algorithm S12

Figure S4. Performance of the TS peak detection algorithm S12

Figure S5. Schematic illustration of the adsorption of proteins to the wall inside a nanopore S13

Figure S6. Example of simulated current traces S13

Figure S7. Analysis of the required time for the TSW algorithm S14

Figure S8. Comparison of the detection rates for the TSW and TS algorithms S14

Figure S9. Comparison of the dwell time and relative current blockades determined by the TS algorithm and the TSW algorithm using simulated data S15

Figure S10 Flowchart illustrating the process of real-time determination of resistive pulses and their classifications from nanopore recordings S16

Figure S11 Probability density function of intra-event current from the simulated traces of five-protein mixtures S17

Figure S12. Comparison of computation time between TSW and TSW-NB algorithms S17

Figure S13 Graphic user interface of the ReverseTrap submodule in PyDAQ S18

Figure S14. Effects of baseline modulation on the determination of protein shape and volume S19

References S19

Supplementary Note 1. Algorithms for calculating mean, median, standard deviation, minimum, and maximum in the sliding windows

Sliding mean and standard deviation

The TSW algorithm calculates the sliding mean using the following equation at each iteration of the program.

$$\overline{x_n} = \overline{x_{n-1}} + \frac{x_n - x_{n-k}}{k} \qquad (1)$$

Here, the $\overline{x_n}$ is the average current in the sliding window at the position of n, and x_n is the current at the position of n. The n is the *nth* point received from the input. The parameter k is the size of the sliding window.

The TSW algorithm calculates the sliding standard deviation using the following equation at each iteration of the program.

$$d_n^2 = d_{n-1}^2 + (x_n - x_{n-k})\,(x_n + x_{n-k} - \overline{x_n} - \overline{x_{n-1}}) \qquad (2)$$

Here, the d_n^2 is the non-normalized variance of the current in the sliding window at the position of n.

Sliding median

The TSW algorithm uses the balanced binary tree (*i.e., Red-Black Tree*)[1] as the sliding window to calculate the sliding median value on the current trace, as shown in **algorithm 1**.

Algorithm 1. Sliding Median

```
Input: timeSeries; Output: medianValues
Initialize redBlackTree as an empty set; Initialize medianValues as an empty array.
For i from 0 to length(timeSeries) – 1
        Insert timeSeries[i] into redBlackTree
        If the size of redBlackTree is greater than windowSize
                Delete redBlackTree[i - windowsize]
        Else
                medianValues[i] = redBlackTree[windowSize/2]
Return
```

Sliding minimum and maximum

The TSW algorithm uses *dequeue* as the sliding window to calculate the sliding *minimum*, and *maximum* value on the current trace, as shown in **algorithm 2**.

```
Algorithm 2. Sliding Minimum, Maximum
Input: timeSeries; Output: minValues, maxValues
Initialize maxque and minque as empty double-ended queues
Initialize medianValues as an empty array
For i from 0 to length(timeSeries) – 1
        While not maxque.empty() and maxque.front()<= i - windowSize
                maxque.pop_front()
        While not minque.empty() and minque.front()<= i - windowSize
                minque.pop_front()
        While not maxque.empty() and timeSeries[maxque.back()]<=timeSeries[i]
                maxque.pop_back()
        While not minque.empty() and timeSeries[minque.back()]>=timeSeries[i]
                minque.pop_back()
        maxque.push_back(i); minque.push_back(i)
        maxValues.append(timeSeries[maxque.front()])
        minValues.append(timeSeries[minque.front()])
Return
```

Supplementary Note 2. Simulation of protein translocation through nanopores

Simulation of intra-event current modulations

The fundamental principle of nanopore-based characterization of protein volume and shape entails deciphering the rotation of a single non-spherical particle as it translocates through a cylindrical nanopore with uniform electric field. The induced current modulation resulting from this rotation can be used to determine the protein shape and volume. The concept draws upon the work of Golibersuch[2], who demonstrated both the theoretical concept and experimental techniques for determining the geometry of red blood cells, which exhibit an oblate shape.[3] As red blood cells pass through and rotate within an electrolyte-filled microchannel, they distort the electric field, resulting in changes in the ionic current that are

directly related to the shape of blood cells. Assuming the object undergoes random rotation along one axis, the corresponding electrical shape factor, γ, can be expressed by Equation 3. Here, γ_v and γ_h are shape factors when the protein's singleton axis (the normal vector of the circle plane) is oriented perpendicular to or in parallel with the nanopore channel, respectively, and θ is the angle between the protein's singleton axis and nanopore cross-section plane.

$$\gamma = \gamma_v + (\gamma_h - \gamma_v)cos^2(\theta) \qquad (3)$$

Proteins undergo frequent collisions by solvent molecules in solutions, resulting in Brownian motion.[4, 5] Brownian motion was elucidated by Einstein with Equation 4. Here, *D (m²/s)* is the diffusion coefficient, η (Pa·s) is the viscosity of the solution, *r (m)* is the radius of the particles, k_B (J/K) is the Boltzmann constant and *T (K)* is the absolute temperature.

The diffusion coefficient, *D*, is related to the hydrodynamic radius of the particle, r_h.[3,9] Assuming that the protein is an ellipsoid, the rotation of a protein can be described by the

$$D = \frac{k_B T}{6\pi\eta r} \qquad (4)$$

$$D_r = \frac{3kT}{16\pi\eta a^3} / (\frac{1 - \frac{1}{m^4}}{\left(2 - \frac{1}{m^2}\right) G(m) - 1}) \qquad (5)$$

rotational diffusion coefficient:

Here, the shape *m (unitless)* is the ratio of the two axes of the ellipsoid, and *a (m)* is the radius of the short axis. *G(m)* is calculated with following equations:

For the oblate:

$$G(m) = \frac{\sqrt{\tan^{-1}\frac{1}{m^2} - 1}}{\sqrt{\frac{1}{m^2} - 1}}$$

For the prolate:

$$G(m) = \frac{1}{\sqrt{1 - \frac{1}{m^2}}} \log_e(m\left(1 + \sqrt{1 - \frac{1}{m^2}}\right))$$

With this rotational diffusion coefficient, we can calculate the angular change of the protein in one dimension as a result of rotational diffusion.

$$\Delta\theta = \sqrt{2D_r\Delta t} \qquad (6)$$

Here, θ represents the angle between the vector of the singleton axis and the plane of the nanopore. Protein orientation in the nanopore affects the electric field distribution, as previously described in Equation 1 for the case of 2D free rotation.[4] In the case of 3D rotation of an ellipsoid of ratio with axes (*a, a, b*) with two independent axes, the shape factor can be described as[3]:

$$\gamma = \gamma_v + (\gamma_h - \gamma_v)(cos(\theta_x)\cos(\theta_y))^2 \qquad (7)$$

We simulate the current induced by protein orientation using an angular random walk approach. The probabilities of moving to θ_x and θ_y direction are equal (50 % for each) when ignoring the influence of the electric field on the dipole moment of proteins. For proteins with non-uniform surface charge, the orientation of the protein will be biased by the electric field. Equation 8 describes the probability of the direction of protein rotation. Here, $P_\pm$ is the probability of the direction of movement at the current position, *E (V/m)* is the electric field intensity, k_B is the Boltzmann constant, *T* is absolute temperature, μ is dipole moment of protiens, and θ represents the angle between the vector of the singleton axis and the plane of the nanopore.[4]

$$P_\pm = \frac{1}{1 + e^{\pm E\mu[\cos(\theta-\Delta\theta) - \cos(\theta+\Delta\theta)]/(2k_BT)}} \qquad (8)$$

Here, *E* is calculated with following equation.

$$E = \frac{V\dfrac{Rl_p}{\pi r_p^2}}{\dfrac{R}{\pi r_p^2} + \dfrac{R}{2r_pl_p}}$$

Simulation of the duration of resistive pulses

The duration of each resistive pulse is simulated as two types: 1. Free translocation events, and 2. adsorption events. The simulation of translocation times starts from a biased random walk[6] of proteins from the *cis* side of the nanopore to the *trans* side. Equation 9 represents a differential form to calculate the movement of the proteins in the nanopore:

$$\Delta x = v_d \Delta t + \sqrt{2D\Delta t} * B \quad (9)$$

Here, the Δx (m) is the updated distance for the protein during a time step Δt. D is the one-dimensional diffusion coefficient of the protein, and B is a random number from a standard Normal distribution. The drift velocity v_d is given by $v_d = u_e E$, where E is the electric field and u_e is the electrophoretic mobility. According to Smoluchowski theory[7], the electrophoretic mobility is defined as $u_e = \varepsilon_r \varepsilon_0 \zeta / \eta$ ($m^2 \cdot s/V$). Adsorption of proteins to the pore wall can occur during the process of translocation. We model the adsorption process using a Markovian reaction, which reveals the distribution of dwell times of adsorption or dissociation, $t_{on,}$ or t_{off} by sampling from an exponential distribution.[8]

$$p(t \mid k_i) = k_i e^{-k_i t} \quad (10)$$

Here, $p(t \mid k_i)$ is the probability density function of lasting time, t, in the *ith* state, given the stochastic rate constant, k_i, for dissociation. **Figure S5** represents the model of protein adsorption during translocation. We calculate the dwell time of free translocation, $t_{d,}$ from the iteration steps using Equation 9 until the proteins leave the channel of the pore. Then, we generate a random number, t_{on}, from the PDF distribution in Equation 8 given k_{on} to determine whether the adsorption occurs by $t_{on} < t$. If the adsorption occurs, the t_{off} generated from the PDF in Equation 10 with giving k_{off} will be the new duration time induced by adsorption.

The event frequency is simply described by an exponential distribution, which has a parameter capture rate constant of k_f (s^{-1}). Besides, we convolve a white noise on the simulation data to simulate the recording noise in nanopore experiments. **Figure S6** represents an example of simulated current as a function of time, showing signals similar to experimental data, including resistive pulses with both short and long dwell times.

Supplementary Note 3. Naïve Bayes classifier for classification of protein mixtures

Given a resistive pulse represented by a current series $x = (x_1, x_2, ..., x_i)$, the classifier calculates the posterior probability of the event belonging to each class C_k (e.g., protein type A and B) using Bayes' theorem. The "naïve" assumption is that all values in x are independent features. This allows the joint likelihood to be factorized into a product of simple probabilities. Therefore, equation 11 represents the posterior probability of the current series during recording:

$$P(C_k \mid x) = \frac{\prod_i P(x_i \mid C_k)\, P(C_k)}{P(x)} \quad (11)$$

Where: $P(C_k \mid x)$ is the posterior probability. The likelihood $P(x_i \mid C_k)$ is derived from the probability density function of the current modulation within events, which can be inferred from experiments using pure protein samples or from the theoretical distribution based on the expected shape and volume. $P(C_k)$ is the prior probability, which can be determined from overall distribution of all protein classes in the mixture.

In our implementation, the probability distributions of likelihood are modeled based on the bimodal distribution as explained by Yusko et al.[3], which are characterized by their

minimum current (*ΔI*$_{min}$), maximum current (*ΔI*$_{max}$) and standard deviation (σ). The equations of current probability density function for oblate and prolate proteins are as follow:

$$P(\Delta I_\gamma) = \frac{1}{A}\cosh\left(\frac{E\mu\left(\sqrt{\frac{\Delta I - \Delta I_{min}}{\Delta I_{max} - \Delta I_{min}}}\right)}{k_B T}\right)\frac{1}{\pi\sqrt{(\Delta I - \Delta I_{min})\,(\Delta I_{max} - \Delta I)}} \quad (S12)$$

$$P(\Delta I_\gamma) = \frac{1}{A}\cosh\left(\frac{E\mu\left(\sqrt{\frac{\Delta I - \Delta I_{max}}{\Delta I_{min} - \Delta I_{max}}}\right)}{k_B T}\right)\frac{1}{\pi\sqrt{(\Delta I - \Delta I_{min})\,(\Delta I_{max} - \Delta I)}} \quad (S13)$$

Consequently, this model assign a label for each resistive pulses by selecting the class with the maximum posterior probability.

Supplementary Note 4. Effects of baseline modulation on the determination of protein shape and volume

To quantify the impact of baseline fluctuation, we calculated the theoretical current blockades and protein volumes under an extreme scenario where the baseline shifts from 14.4 nA to 15.6 nA, corresponding a pore diameter shift from 19.5 to 20.5 nm, in a nanopore with a diameter of 20 nm and a length of 30 nm. As shown in **Supplementary Figure S14**, the estimated protein volume varies only within a range of ~13 nm3 (~10 %) in response to this baseline shift, indicating minor uncertainty. Furthermore, this baseline variation induces a change of only ~15 pA in the current blockade (*ΔI*), which is small compared to the typical system noise of 50–100 pA. Since the protein shape is derived from fitting the probability density function of current blockades, the influence of baseline shifts on estimated protein shape is also negligible.

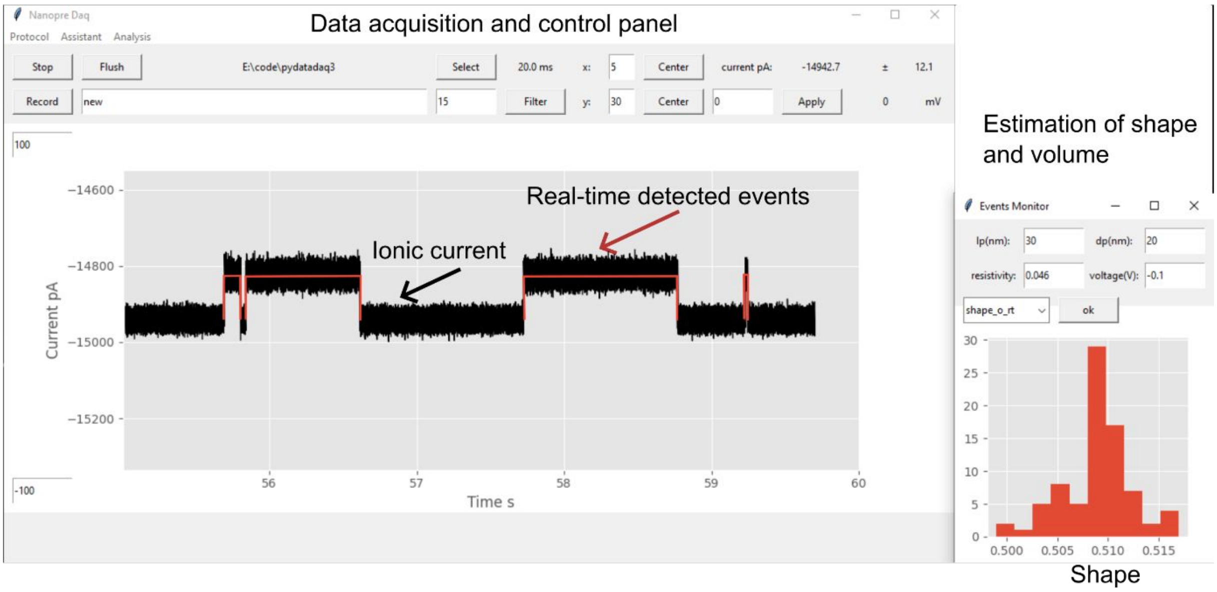

Figure S1. Screenshot of the data acquisition and real-time estimation of protein using PyDAQ. The graphical user interface controls the data acquisition, low-pass filter, visualization, resistive pulse detection, and protein estimation. The main plot shows the ionic current (in pA) over time, with detected resistive pulses shown as thin red lines in real time. The histogram on the right provides real-time updates that instantly estimate the shape and volume of the detected proteins.

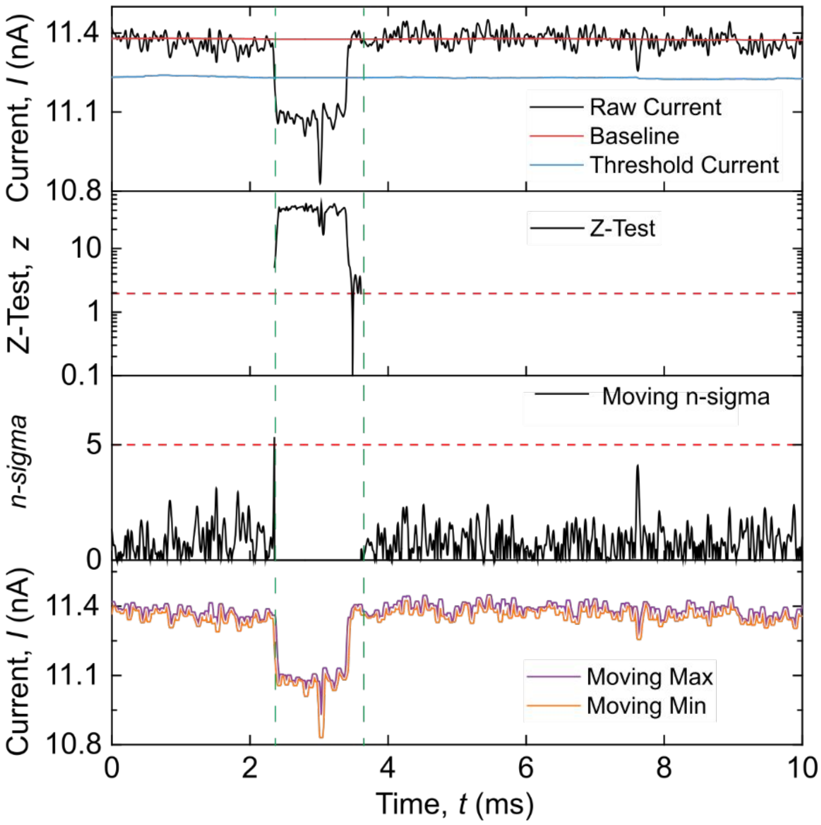

Figure S2. Example current trace demonstrating the changing of internal variables calculated by the TSW algorithm during the detection of a resistive pulse. The first panel shows a representative ion current trace containing a single resistive pulse. The blue line represents the continuously updated threshold current for determining the start of the resistive pulse during the TSW processing. The red line represents the moving baseline during the TSW processing. The second panel shows the z-score (*z-test*) to determine the end of the resistive pulse, with the red dashed line indicating a significance threshold of 1.96. The third panel displays the moving *n*-sigma used to determine the start of the resistive pulse, with a threshold of 5 shown by the red dashed line. The bottom panel shows the moving minimum and maximum currents within the statistical window w_2.

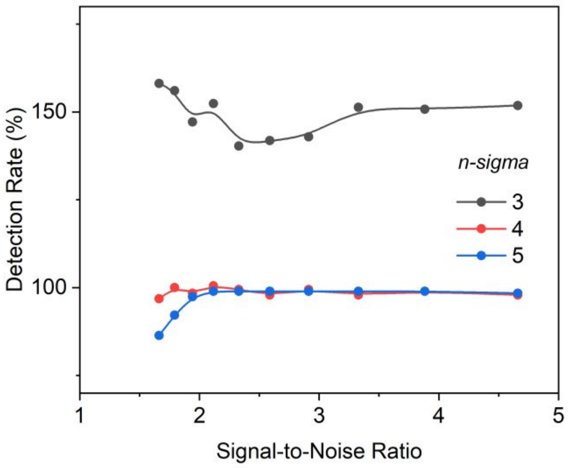

Figure S3. Effects of *n-sigma* parameter and signal-to-noise ratio of resistive pulses on the performance of the TSW algorithm. The analysis employs the simulated datasets from the translocation of a mixtures of five proteins used in **Figure 6**.

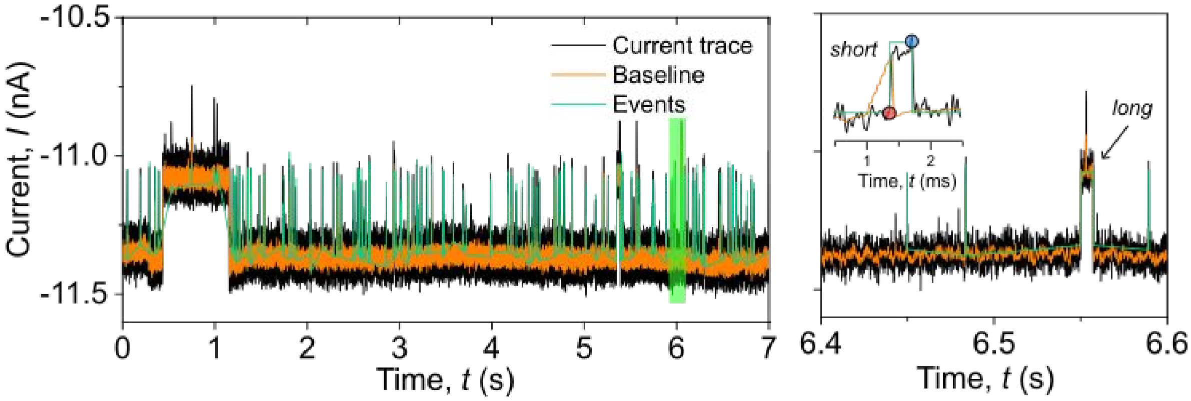

Figure S4. Performance of the TS peak detection algorithm. The left panel represents an experimental recording current trace (black line), its baseline (orange line), and resistive pulses (green line) detected using the TS algorithm. The right panel zooms in on the current trace between 5.9 ~ and 6.1 s from the left panel (light green shading). The inset represents a short resistive pulse, its baseline current (orange line), and the start (red circle) and end (blue circle) of the resistive pulses.

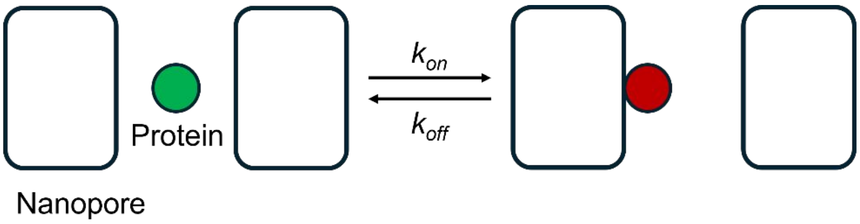

Figure S5. Schematic illustration of the adsorption of proteins to the wall inside a nanopore. The green circle represents the proteins that translocate through a nanopore without adsorption, and the red circle represents the proteins that undergo adsorption. The adsorption process can be described as a Markovian reaction.[8] The parameters k_{on} and k_{off} represent stochastic rate constants of the adsorption or dissociation between protein and nanopore surface.

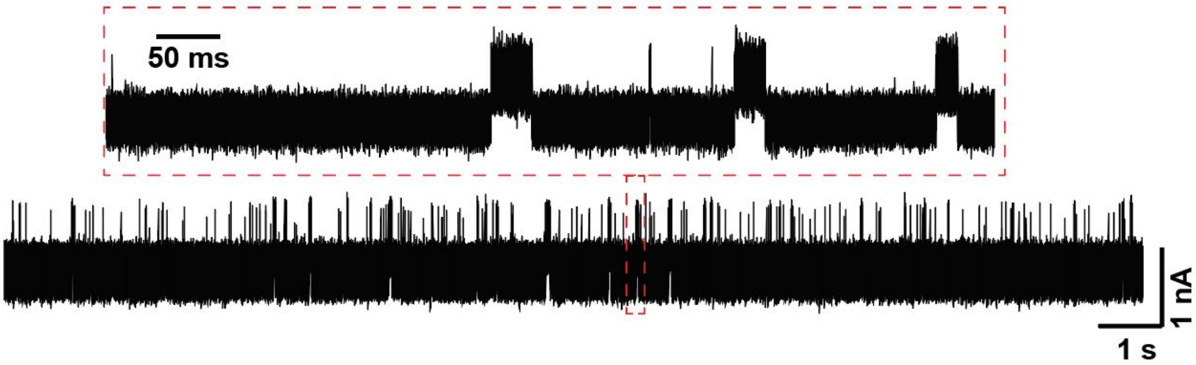

Figure S6. Example of simulated current traces. The trace represents 20 seconds of simulated translocations of oblate particles through a nanopore with a diameter of 20 nm and a length of 30 nm. The red dashed box represents a zoom-in current trace, showing either long resistive pulses from simulated adsorption or short resistive pulses from free translocation of proteins.

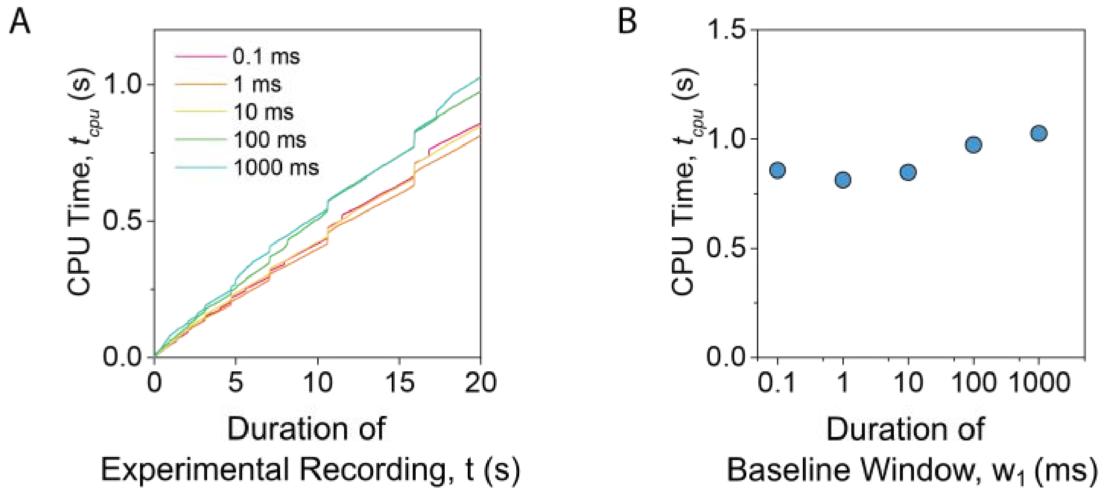

Figure S7. Analysis of the required time for the TSW algorithm. A. Cumulative CPU computation time as a function of duration of experimental recording. Colored curves represent the analysis using different durations of the baseline window, w_1. **B.** Cumulative CPU time as a function of the duration of the baseline window w_1 for analyzing 20 s data at 500 kHz sampling rate. The CPU time does not show significant changes over the size of the baseline window w_1.

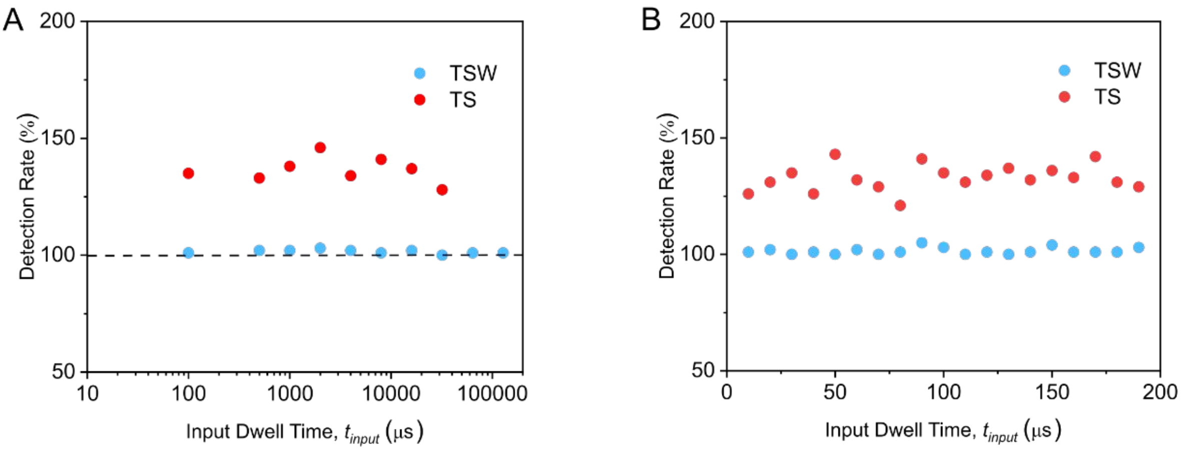

Figure S8. Comparison of the detection rates for the TSW and TS algorithms. A, B. Detection rate as a function of input dwell time for the TSW (blue) and TS (red) algorithms. The analysis used the same datasets as those used in **Figure 4 C, E**. The detection rate is defined as the ratio of the number of detected resistive pulses to the number of simulated resistive pulses. A detection rate exceeding 100% indicates the detection of false positive events.

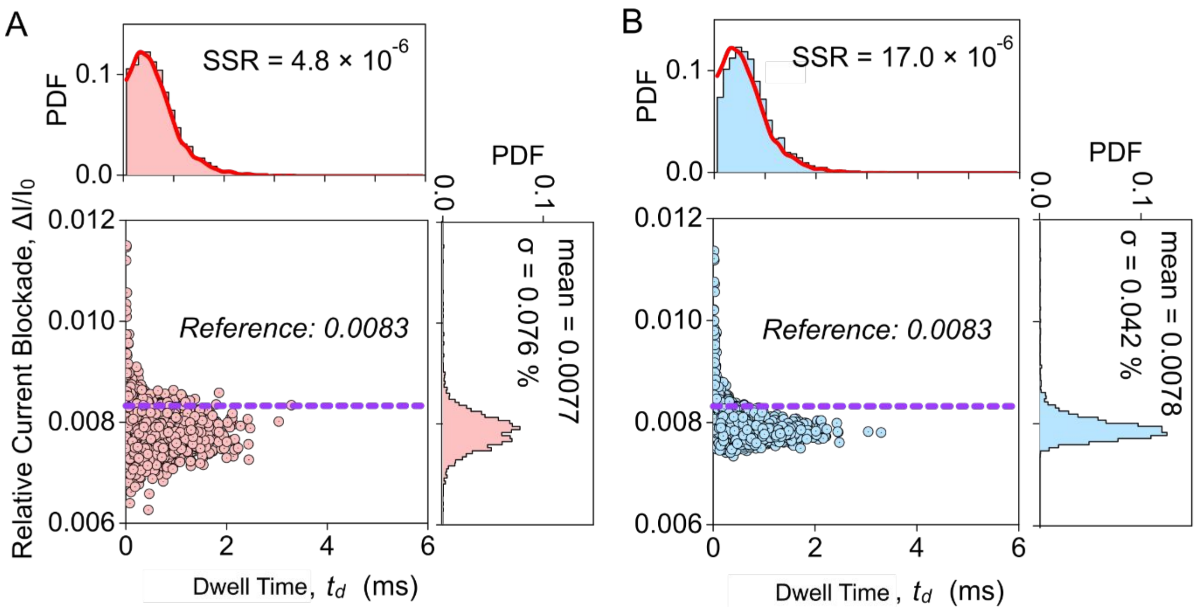

Figure S9. Comparison of the dwell time and relative current blockades determined by the TS algorithm (A) and the TSW algorithm (B) using simulated data (Supplementary Note 2). Each panel shows a 2D scatter plot of pulse relative current blockade and dwell time, with marginal histograms for each parameter. The purple dashed line in each scatter plot marks the reference value of the relative current blockades (0.0083). The red curves overlaid on the histograms of dwell time represent the ground truth probability density function (PDF) used in the simulation. The sum of squared residuals (SSR) shows the agreement between the ground truth and the determined dwell time by the TS or TSW algorithm.

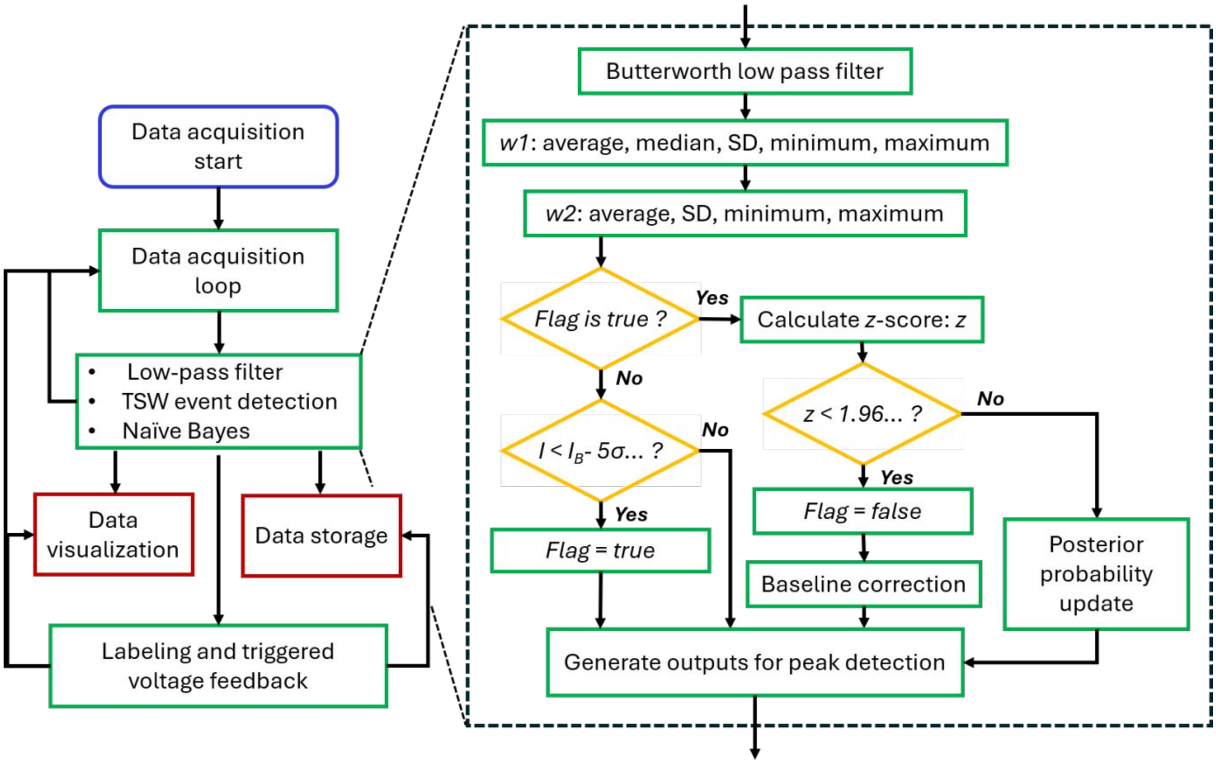

Figure S10 Flowchart illustrating the process of real-time determination of resistive pulses and their classifications from nanopore recordings. Data is collected from a data acquisition card every 20 ms in each loop. Concurrently, data are processed in real time via a low-pass Butterworth filter and the TSW-NB algorithm (inset). Detected events are visualized in real time and stored on disk by a different task process. Simultaneously, the program determines the voltage to be applied based on the continuous stream of protein classification labels.

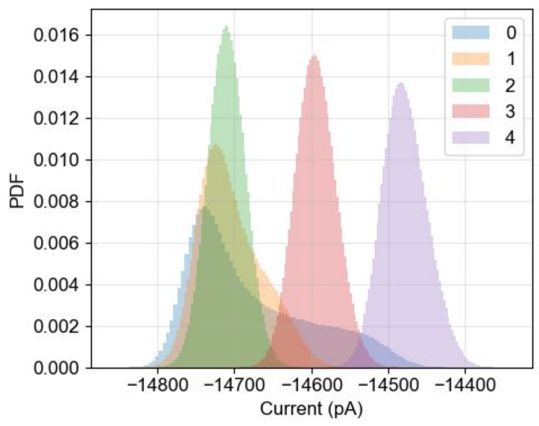

Figure S11 Probability density function of intra-event current from the simulated traces of five-protein mixtures. Proteins are defined by shape (m) and volume (V): 0 (m 0.3, V 150 nm^3), 1 (m 0.5, V 150 nm^3), 2 (m 0.8, V 150 nm^3), 3 (m 0.8, V 225 nm^3), and 4 (m 0.8, V 300 nm^3). The simulation was performed on a nanopore with diameter of 20 nm and length of 30 nm in 2M KCl with an applied voltage of -0.1 V. The signal was sampled at 500 kHz sampling rate with a 50 kHz low-pass filter and added with 50 pA Gaussian noise.

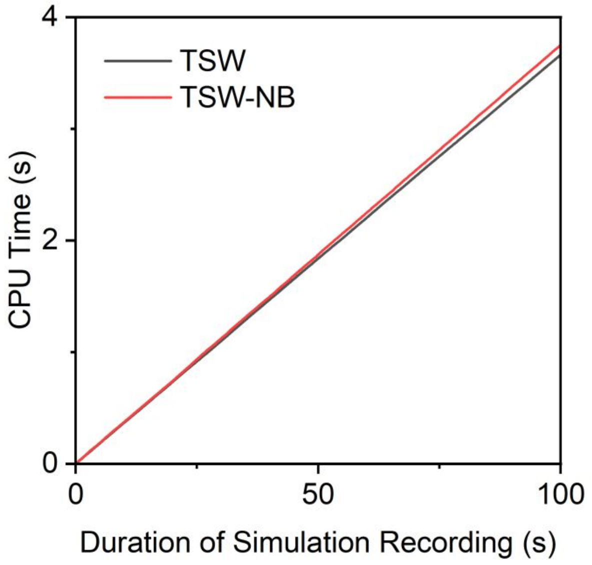

Figure S12. Comparison of computation time between TSW and TSW-NB algorithms. During a 100 s recording at a 500 kHz sampling rate, the total cumulative CPU computation time for TSW-NB slightly increased to 3.75 s, compared to 3.66 s for original TSW algorithm.

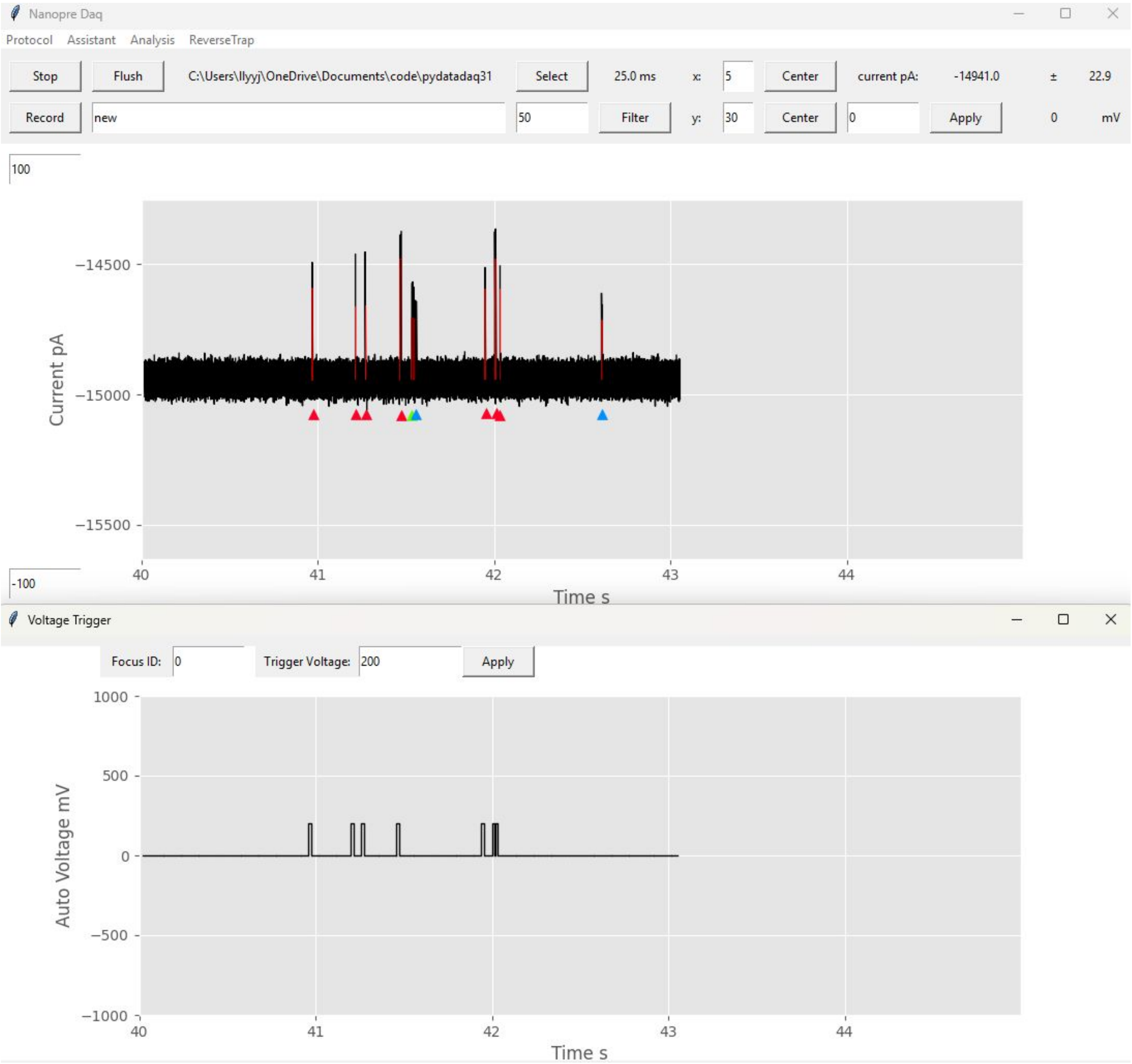

Figure S13 Graphic user interface of the ReverseTrap submodule in PyDAQ. The screenshot illustrates the real-time protein classification and voltage triggering. The main plot shows the ionic current (in pA) over time, with resistive pulses marked and classified in real time, with thin read lines marking the detected pulses and colored solid triangles indicating their classifications. The bottom panel provides voltage control to instantly apply a strong reverse voltage for specific protein types (indicated here by red solid triangles) during recordings.

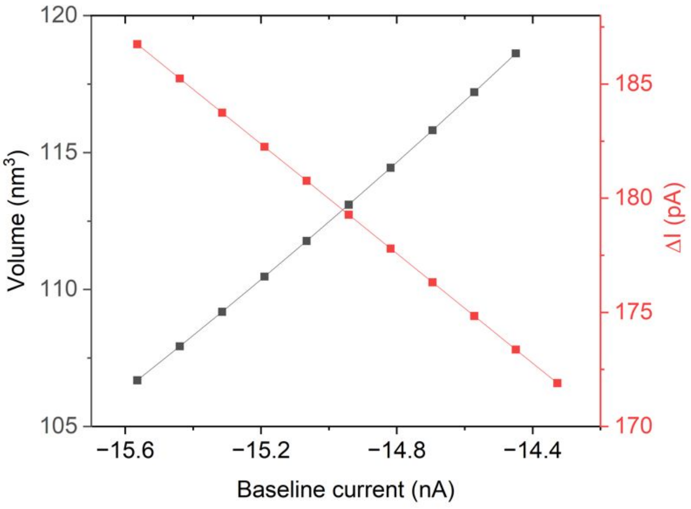

Figure S14. Effects of baseline modulation on the determination of protein shape and volume. The calculation was performed using a nanopore with a diameter of 20 nm and a length of 30 nm at an applied potential of -100 mV, the protein was modelled with a diameter of 6 nm and a shape factor of m = 1.0. Since protein shapes are derived from fitting the probability density function of current blockades, the influence of baseline shifts on the estimated protein shape is negligible, given the small change of ~15 pA in the current blockades, ΔI.

References

1. Knuth, D. E., *The Art of Computer Programming*. Addison-Wesley: 2005.

2. Golibersuch, D. C., Observation of Aspherical Particle Rotation in Poiseuille Flow Via Resistance Pulse Technique .2. Application to Fused Sphere Dumbbells. *J Appl Phys* **1973,** *44* (6), 2580-2584.

3. Yusko, E. C.; Bruhn, B. R.; Eggenberger, O. M.; Houghtaling, J.; Rollings, R. C.; Walsh, N. C.; Nandivada, S.; Pindrus, M.; Hall, A. R.; Sept, D.; Li, J. L.; Kalonia, D. S.; Mayer, M., Real-time shape approximation and fingerprinting of single proteins using a nanopore. *Nat Nanotechnol* **2017,** *12* (4), 360-367.

4. Dickinson, E., Brownian Dynamics with Hydrodynamic Interactions - the Application to Protein Diffusional Problems. *Chem Soc Rev* **1985,** *14* (4), 421-455.

5. Albrecht, T., Single-Molecule Analysis with Solid-State Nanopores. *Annu Rev Anal Chem* **2019,** *12*, 371-387.

6. Freund, Y.; Opper, M., Drifting games and Brownian motion. *J Comput Syst Sci* **2002,** *64* (1), 113-132.

7. Saffman, P. G., Happel,J - Low Reynolds Number Hydrodynamics with Special Applications to Particulate Media. *Nature* **1966,** *212* (5066), 1032-&.

8. Spies, M.; Chemla, Y. R., METHODS IN ENZYMOLOGY Single-Molecule Enzymology: Fluorescence-Based and High-Throughput Methods PREFACE. *Single-Molecule Enzymology: Fluorescence-Based and High-Throughput Methods* **2016,** *581*, Xvii-Xviii.

9. Dubin, Stuart B .Measurement of the Rotational Diffusion Coefficient of Lysozyme by Depolarized Light Scattering: Configuration of Lysozyme in Solution. *Journal of Chemical Physics*, **1971**, *54(12)*, 5158-5164.
